# New Insights into Blood Circulating Lymphocytes in Human *Pneumocystis* Pneumonia

**DOI:** 10.3390/jof7080652

**Published:** 2021-08-11

**Authors:** Eléna Charpentier, Catherine Marques, Sandie Ménard, Pamela Chauvin, Emilie Guemas, Claire Cottrel, Sophie Cassaing, Judith Fillaux, Alexis Valentin, Nicolas Blanchard, Antoine Berry, Xavier Iriart

**Affiliations:** 1 Department of Parasitology-Mycology, Toulouse University Hospital, 31059 Toulouse, France; chauvin.p@chu-toulouse.fr (P.C.); guemas.e@chu-toulouse.fr (E.G.); claire.cottrel@hotmail.fr (C.C.); cassaing.s@chu-toulouse.fr (S.C.); fillaux.j@chu-toulouse.fr (J.F.); alexis.valentin@univ-tlse3.fr (A.V.); berry.a@chu-toulouse.fr (A.B.); 2 Infinity, Inserm, CNRS, University of Toulouse III, 31024 Toulouse, France; catherine.marques@inserm.fr (C.M.); sandie.menard@inserm.fr (S.M.); nicolas.blanchard@inserm.fr (N.B.)

**Keywords:** *Pneumocystis jirovecii*, pneumocystosis, immunophenotyping, lymphocyte, T helper, B lymphocytes

## Abstract

The host lymphocyte response is decisive in *Pneumocystis* pneumonia (PCP) pathophysiology but little is known of the specific roles of lymphocyte subpopulations in this fungal infection. Peripheral NK, NKT, B, TCD4+ and TCD8+ subpopulations were compared by immunophenotyping between 20 patients diagnosed with PCP (PCP(+)] and 20 uninfected immunosuppressed patients (PCP(−)). Among PCP(+) subjects, the lymphocyte populations were also compared between surviving and deceased patients. Low B cell count (<40 cells/µL) was more frequent in PCP(+) than in PCP(−) patients (*p* = 0.03), while there was no difference for the TCD4 count. Among the PCP(+) group, the 7 deceased patients had lower Th1 (*p* = 0.02) and Tc1 (*p* = 0.03) populations, higher Th2 response (*p* = 0.03), higher effector TCD8 (*p* < 0.01), lower central memory TCD8 (*p* = 0.04) and reduced NK cells (*p* = 0.02) compared with the 13 survivors. Th1/Th2 ratio < 17, CD8 Tc1 < 44%, effector TCD8 < 25%, central memory TCD8 < 4%, NK cells < 50 cells/µL and total lymphocytes < 0.75 G/L were associated with a higher risk of mortality (*p* = 0.003, *p* = 0.007, *p* = 0.0007, *p* = 0.004, *p* = 0.02 and *p* = 0.019, respectively). The traditional analysis of TCD4 and TCD8 populations may be insufficient in the context of PCP. It could be completed by using B cells to predict the risk of PCP, and by using lymphocyte subpopulations or total lymphocyte count, which are easy to obtain in all health care facilities, to evaluate PCP prognosis.

## 1. Introduction

*Pneumocystis* pneumonia (PCP) is an opportunistic fungal disease that induces severe hypoxia and potentially life-threatening respiratory distress syndrome. It remains one of the most frequent AIDS-defining infections, and its incidence is increasing among non-HIV subjects in parallel to the growing use of immunosuppressive drugs, including corticosteroids [1,2]. Despite adapted antimicrobial treatment, PCP mortality is estimated at 20–30%, with variations depending on patients’ underlying diseases [1,3,4].

The pathophysiology of this fungal pneumonia remains poorly understood; however, it is generally accepted that the host’s unsuitable and exaggerated inflammatory response is largely responsible for the lung lesions and PCP respiratory symptoms. Lymphocytes, in particular, play a critical role in the disease development because they are needed to clear the fungus but are also paradoxically implicated in the lung tissue damage [5,6].

TCD4 lymphocytes are key cells for the defense against *Pneumocystis*, and a count < 200 cells/µL greatly increases the risk of PCP, mostly in HIV-positive patients [7]. TCD8 lymphocytes have a controversial role in PCP. Some murine studies found that TCD8 can participate in fungal clearance in the absence of TCD4 [8,9], while others report a pathological effect on alveoli tissue in PCP [5].

TCD4 and TCD8 populations, however, include various subpopulations with specific roles that could have different impacts on the disease. Despite several murine experiments on lymphocyte response during PCP, the TCD4 subpopulations (including Th1, Th2, Th17 and regulatory T cells) that are actually required to eliminate *Pneumocystis jirovecii* have still not been clearly identified [8,10,11]. As for clinical studies on PCP in humans, these are essentially limited to a retrospective analysis of the TCD4 and TCD8 populations without further investigations into their subpopulations. Recently, Zhang et al. explored T lymphocyte subpopulations in patients with PCP and found a potential protective role of the CD4 Th1 and cytotoxic CD8 Tc1 subsets [12]. These results are, however, limited to a specific population of patients with inflammatory or autoimmune diseases. Furthermore, clinical studies rarely focus on B lymphocytes or NK cells, which could have a role in PCP according to murine experiments [13,14]. Considering the severity of PCP, a better understanding of its pathophysiology and the determination of suitable risk factors and prognostic factors for all patients are required.

In this context, we performed a pilot prospective clinical study that aimed to describe both the TCD4 and TCD8 subpopulations as well as peripheral blood B cells, NK cells and NKT cells in PCP and to compare those populations with uninfected immunosuppressed patients. A second objective was to compare the levels of lymphocyte subpopulations in patients with PCP according to the outcome of the disease (survivors versus deceased patients).

## 2. Patients and Methods

### 2.1. Subject Inclusion and Sample Collection

The study was carried out prospectively from August 2016 to September 2018 in Toulouse University Hospital. Patients included in the PCP-positive (PCP(+)) group were adults diagnosed with PCP based on three combined arguments. First, all patients had a positive *P. jirovecii* qPCR in a respiratory sample (performed as previously described by Fillaux et al., 2008 [15]) with a high fungal load (qPCR cycle threshold (Cq) < 27.5 cycles for bronchoalveolar lavage or Cq < 24 cycles for sputum and tracheal aspiration). Secondly, PCP(+) patients all had PCP-compatible symptoms (i.e., dyspnea, fever and/or cough). Thirdly, they all had PCP-compatible imaging (ground glass opacities or abnormalities of interstitial lung tissue). All PCP(+) patients received curative anti-*Pneumocystis* treatment and had a concomitant white blood cell analysis (on the day of the diagnosis or within the next three days). The day of diagnosis was defined as the day a positive *P. jirovecii* qPCR was found. The PCP-negative (PCP(−)) group included the first patients with a negative *P. jirovecii* PCR that chronologically followed a PCP(+) patient included in the study, along with the subsequent requirements of a concomitant white blood cell analysis and a known cause of immunosuppression. In the PCP(+) group, the patient’s outcome (death or survival) was evaluated at 2 months post-diagnosis.

Blood samples of all included patients were obtained from physicians’ prescriptions for white blood cell analysis, and clinical data were collected from a review of the patients’ medical charts. In accordance with the French public health law [16], this protocol did not require approval from an ethics committee and was exempt from formal informed consent.

Peripheral blood mononuclear cells (PBMC) of 20 anonymous adult male and female healthy blood donors (no known immunosuppression condition) were also analyzed in order to estimate normal reference values of the different lymphocyte subpopulations.

### 2.2. Leukocyte Count and Differential

Leukocyte counts and differentials were analyzed at the hematology laboratory of Toulouse University Hospital with a Sysmex XN cytometer (Sysmex Corporation, Kobe, Japan). Leukocyte counts and differentials were not performed on anonymous healthy control subjects for whom only PMBC were available.

### 2.3. PBMC Isolation and Lymphocyte Immunophenotyping

PBMC were collected from the patients’ EDTA blood samples using a lymphocyte separation medium (Ficoll-Human density 1.077g/mL, PAN Biotech, Aidenbach, Germany). Harvested cells were cryopreserved in a 9:1 medium of fetal calf serum (Gibco) and dimethyl sulfoxide (Sigma-Aldrich, St. Louis, MO, USA) until immunophenotyping analysis.

Lymphocyte subpopulations were determined with the following panel of 14 antibodies (BD Biosciences, Franklin Lakes, NJ, USA): CD3-APC-H7, CD4-BV786, CD8α-BV711, CD19-BV510, CD25-BV421, CD45RA-PE-Cy7, CD56-BV605, CD127-AF647, CD183-BB700, CD194-BV650, CD196-PE-CF594, CD197-BB515, Foxp3-PE and a viability marker (FVS-APC R700). Frozen PBMC were thawed, and a total of 500,000 cells were labeled after saturation with human serum. Cells were successively stained with the viability dye, the surface antibodies and, finally, with FoxP3 after cell fixation and permeabilization with Transcription Factor Buffer (BD Biosciences). For all experiments, acquisition was performed using LSR-Fortessa flow cytometer (BD Biosciences) and data were analyzed using Flowjo software (version 10). The gating strategy is detailed in the Supplementary Data section (Supplementary Figure S1). In order to avoid uncertain results, the TCD4 and TCD8 subpopulations were not analyzed when there were fewer than 500 events for TCD4 or TCD8 cells.

### 2.4. Statistical Analysis

Comparison of the patient characteristics and qualitative analysis of the lymphocyte subpopulations between the different groups of interest were performed using the chi-squared test or Fisher’s exact test, as appropriate. The distribution of leukocyte and lymphocyte populations was analyzed using the Mann–Whitney U test or Kruskal–Wallis test and Dunn’s test for comparison with reference values. Relationships between B lymphocyte count and the cumulative dose of corticosteroid were analyzed by Spearman rank-order correlation test. A multivariate logistic regression analysis was conducted to identify the variables that exhibited independent associations with the presence of PCP. Relative risks were obtained by odds ratios (OR) with 95% confidence intervals (95% CI). Survival analyses were performed with the Kaplan–Meier analysis and compared with the Mantel–Cox test. All analyses were performed on GraphPad Prism version 5.0 software (GraphPad) except for the ROC curves, which were done using R software with the “pROC” package [17] and compared with the DeLong test. The differences were considered significant when *p* < 0.05, and *p*-values were then underlined in the tables.

## 3. Results

### 3.1. Patient Characteristics

Between 2016 and 2018, 20 patients were included in the PCP(+) group and 20 patients in the PCP(−) group. All demographic and clinical data are detailed in Table 1. There was no significant difference in age, sex, immunosuppressive conditions or co-infections (including active CMV infection) between the PCP(−) and PCP(+) groups A large proportion of patients in the two groups received corticosteroids (70% in the PCP(+) group and 40% in the PCP(−) group, *p* = 0.06). The cumulative dose of corticosteroids over two-months was significantly higher in PCP(+) patients (*p* = 0.04). The number of patients receiving a high cumulative dose over the 2 months preceding PCP (>3000 mg of prednisone or equivalent) was also significantly higher in PCP(+) group (*p* = 0.05). As expected, there were more patients with dyspnea (*p* = 0.01), fever (*p* = 0.01), oxygen requirements (*p* = 0.01) and PCP-compatible imaging (*p* < 0.01) in the PCP(+) group.

### 3.2. Lymphocyte Subpopulations in PCP-Positive versus PCP-Negative Subjects

Lymphocyte populations and subpopulations were analyzed for all patients except for the TCD8 subpopulations of one PCP(+) patient and both TCD4 and TCD8 subpopulations of two PCP(−) patients because of a low number of analyzable cells (<500) for TCD8 and TCD4 (previously fixed threshold). All data are presented in Table 2.

Between the PCP(+) and PCP(−) groups, there was no significant difference in the counts or proportions of total lymphocytes, total TCD4 or for the usual TCD4 threshold of 200 cells/µL (Table 2). The TCD4 and TCD8 subpopulations and NK cells did not differ either. However, more patients in the PCP(+) group had low B lymphocyte counts (<40 cells/µL) compared with the PCP(−) patients (*p* = 0.03), and the total TCD8 proportion tended to be higher in the PCP(+) patients (*p* = 0.06).

As corticosteroid therapies are known to potentially induce a reduction of B cell responsiveness, the relation between these two parameters were evaluated. In our study, there was no correlation between B lymphocyte count and the cumulative dose of corticosteroid (ρ= −0.17; *p* = 0.294, Spearman rank-order test). Moreover, a multivariate logistic regression analysis including these two parameters has shown that B cell count was an independent risk factor for PCP (*p* = 0.046, OR: 4.301, 95% CI: 1.026–18.028 for B lymphocytes < 40 cells/µL; *p* = 0.052, OR: 1.000, 95% CI: 0.999–1.001 for a 2-month cumulative dose of corticosteroid).

An analysis excluding the HIV-infected patients (4 PCP(+) and 4 PCP(−)), who generally have a different PCP clinical course, was performed (data not shown) and gave substantially the same results with a difference in the B cell counts below 40 cells (*p* = 0.03) and the CD8 proportion (*p* = 0.05).

The values of the lymphocyte subpopulations from the control group (healthy people) and their comparison with the PCP(+) and PCP(−) groups are detailed in the Table 2.

### 3.3. Lymphocyte Subpopulations in Deceased Subjects versus Survivors

Among the PCP(+) group, 13 (65%) patients survived, whereas 7 (35%) patients died of respiratory failure. The two groups showed no significant difference in demographics, immunosuppressive conditions or therapies, nor in co-infections. All results are detailed in Table 3.

The comparison of the lymphocyte populations and subpopulations revealed several differences (Table 3). The total lymphocyte and NK cell counts in deceased patients were lower than those in survivors (*p* = 0.05 and *p* = 0.02, respectively).

Total TCD4 lymphocytes were not significantly different between the two groups, but deceased patients had a lower Th1 subpopulation (CD4+ CCR4− CXCR3+ CCR6−), higher Th2 proportion (CD4+ CCR4+ CXCR3- CCR6) and lower Th1/Th2 ratio (*p* = 0.02, *p* = 0.03 and *p* = 0.01, respectively).

Concerning TCD8 lymphocytes, the effector cells proportion was higher in the deceased group (*p* < 0.01), whereas the count of central memory TCD8 (CM TCD8) was lower (*p* = 0.04). Cytotoxic CD8 Tc1 cells (CD8+ CXCR3+) were lower in the deceased group, for the global non-naive TCD8 cells (*p* = 0.03) and for the TCD8 subpopulations (effector Tc1 *p* = 0.04, effector memory Tc1 *p* = 0.003 and CM TCD8 Tc1 *p* = 0.04; data not shown).

Overall, the deceased patients had a reduced Th1/Tc1 pro-inflammatory profile, which was associated with a reduction of NK cells and a higher proportion of non-cytotoxic effector TCD8 cells.

An analysis focused on the 16 HIV-negative patients showed the same results, with a decrease in statistical power due to a smaller number of patients (Th1/Th2 ratio (*p* = 0.02), Th1 (*p* = 0.07), Th2 (*p* = 0.06), NK cells (*p* = 0.02), effector TCD8 (*p* = 0.01), CM TCD8 (*p* = 0.07), Tc1 CD8 (*p* = 0.08)).

### 3.4. Lymphocyte Predictive Values of PCP Outcome

We questioned whether the different levels of lymphocyte populations between survivors and deceased patients could predict the risk of mortality in patients affected with PCP. ROC curves were performed to determine the best threshold for each lymphocyte population. The obtained thresholds were Th1/Th2 ratio < 17, Tc1 CD8 proportion < 44%, effector TCD8 proportion > 25%, NK cells < 50 cells/µL and lymphocyte count < 0.75 G/L. With those thresholds, the negative predictive values (NPV) were higher than 90% for all parameters, and the positive predictive values (PPV) were all around 70%. All NPV, PPV, sensitivities, specificities and areas under the curve (AUC) are detailed in Table 4 (ROC curves are shown in Supplementary Figure S2). Although the total lymphocyte count had the lowest AUC (76.8% vs. 93.1% or 88.1% for the TCD8 effector proportion and Th1/Th2 ratio, respectively), there was no significant difference when comparing all AUCs (Supplementary Table S1).

**Figure 1:**
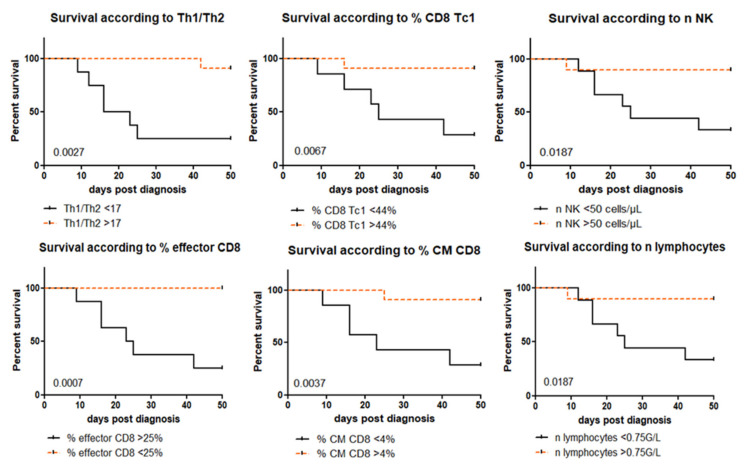
Kaplan–Meier survival curves. *p*-values are added in the lower left corner of the graphs (Mantel–Cox test).

Kaplan–Meier survival curves were performed to compare the mortality over and below the previously fixed thresholds. There were significant differences in mortality according to the Th1/Th2 ratio, CD8 Tc1 proportion, effector TCD8 proportion, CM TCD8, NK cell counts and lymphocyte counts (*p*-values of 0.003, 0.007, 0.0007, 0.004, 0.02 and 0.019, respectively) as shown in Figure 1. Interestingly, a lymphocyte count below 0.75 G/L was associated with a reduced Th1/Th2 ratio and Tc1 CD8 proportion (Supplementary Figure S3).

## 4. Discussion

The host immune response is decisive in *Pneumocystis* infections as it can eliminate the fungus in immunocompetent hosts, but it also causes pulmonary lesions and respiratory symptoms of PCP in immunocompromised hosts. In our study, lymphocyte populations and subpopulations were compared between 20 PCP(+) and 20 PCP(−) patients, and also among PCP(+) subjects between the 13 survivors and the 7 deceased patients.

When comparing the PCP(+) and PCP(−) groups, we observed no significant differences for the TCD4, TCD8 or NK populations. The classical TCD4 count < 200 cells/µL was not associated with a greater risk of PCP in our study, but this major threshold is not as predictive for HIV-negative patients [3,7], who represented 80% of included PCP(+) patients. Other parameters, such as CMV reactivation or age [18], which are classical risk factors for some immunosuppressive conditions such as solid organ transplanted patients, were not significantly different in this study, and this could be related to the diverse causes of immunosuppression in this study.

In contrast, we observed that very low B cell counts (<40 cells/µL) were more frequently associated with PCP(+) than PCP(−) patients. A decreased B cell count has already been described in HIV-negative patients with PCP [19]. From these data, it is not possible to conclude whether the reduction of B lymphocyte preceded the infection or was a consequence (direct or indirect) of PCP. However, there are some arguments indicating that low B lymphocytes could be a risk factor of PCP. A specific deficit of B cells is sufficient for inducing PCP in mice [13,20]. In humans, anti-CD20 monoclonal antibodies (such as rituximab) are potential risk factors for PCP [21]. Also, the very frequent corticosteroid therapies are also known to induce, among other things, a reduction of B cell responsiveness [22,23], as observed in a murine experiment of PCP [24]. In our study, PCP(+) patients had high cumulative corticosteroid doses more frequently, but there was no direct correlation between B cell counts and the cumulative dose of corticosteroids received. Moreover, B cell count appeared to be an independent risk factor of PCP as shown by multivariate logistic regression analysis. These results might possibly be explained by the multiple immunosuppressive therapies received by the patients which could impact B cell count. Nonetheless, these results are in line with the growing interest in B lymphocytes in the context of PCP. Their quantification may be an interesting complement to TCD4 monitoring for PCP risk assessment, especially in HIV-negative patients for whom TCD4 analysis appears unsatisfactory.

In a further step, the lymphocyte populations were compared among the PCP(+) group and between the survivors and deceased patients. As in previous studies, we found a lower global lymphocyte count in the deceased patients [25,26]. Interestingly, the other differences between survivors and deceased patients did not concern TCD4 or TCD8 populations as in previous reports [25,27], but their subpopulations and NK cells, which are not usually explored in PCP clinical studies.

Deceased patients had lower Th1 and Tc1 responses, a higher Th2 profile associated with a higher proportion of effector TCD8, a lower number of CM TCD8 cells and a reduction of NK cell counts. These results suggest a protective role of the pro-inflammatory Th1 profile in PCP, which induces a strong TCD8 response. It concurs with previous rodent studies that reported an improvement of PCP when adding interferon gamma (IFNγ) by aerosol or via an adenovirus [8,28]. In humans, Zhang et al. also observed in a recent clinical study that both CD4 Th1 and CD8 Tc1 proportions were negatively correlated with PCP severity [12]. The controversy about the beneficial or deleterious effects of TCD8 on PCP could be explained, as previously suggested, by the different effects of specific TCD8 subpopulations, underlining the importance of their analysis [8,29]. Hence, highly polarized Tc1 CD8 could play a part in *Pneumocystis* *sp.* elimination. They were shown to induce increased *Pneumocystis* lysis by macrophages in vitro [29]. Conversely, non-cytotoxic effector TCD8 could be deleterious in PCP. Moreover, between survivors and deceased patients, the difference in memory cells, which are generally more efficient in eliminating their specific pathogens, exclusively concerned TCD8 and not TCD4; this emphasizes the role of the TCD8 and Tc1 response in PCP over the Th2 humoral response. Ruan et al. have already described better *Pneumocystis* elimination when memory TCD8 were increased by the addition of IL-7 in CD4-depleted mice [30].

Regarding NK cells, which, to date, have been poorly evaluated in PCP, a low blood count has already been associated to a higher risk of mortality in a recent clinical study on PCP in HIV-negative patients [27]. A murine study also reported higher *P. murina* lung loads in NK-depleted-mice [14]. Low NK cell counts could be associated with the lower Th1/Tc1 profile because NK cells are IFNγ-producing cells. However, their protective role in PCP could also be related to their contribution to antigen-presenting cell stimulation and/or to their cytotoxic activity [14].

The causes of the Th1/Tc1 deficit or the reduced number of CM TCD8 observed in deceased patients were not determined in our study. For instance, they could be related to underlying diseases, therapeutics, specific viral or bacterial coinfections or to a *P. jirovecii* strain. In particular, bacterial coinfections tended to be more frequent in non-survivors of the PCP(+) group (*p* = 0.07). The effect of these coinfections on lymphocyte populations cannot be formally ruled out given the small number of patients. Further evaluations are required to identify host or *Pneumocystis* parameters that contribute to the differences of the TCD4 and TCD8 profiles.

Finally, ROC curves and Kaplan–Meier analyses made it possible to determine thresholds for the lymphocyte count, Th1/Th2 ratio, Tc1 proportion, effector TCD8 proportion and NK cell count to differentiate between surviving and deceased patients. These criteria, including lymphocyte counts (<0.75 G/L), which are easy to obtain in all health care facilities, could help physicians predict the outcome of PCP. Evaluation of the mortality risk could help physicians to adapt patient care (administration route of drugs targeting *Pneumocystis*, oxygen therapy, addition of corticosteroids) considering the potentially severe outcome of the infection. The results characterizing these thresholds (especially NPV and PPV) are performed on a limited number of patients and need to be confirmed on larger cohorts.

This pilot study aimed to evaluate the circulating lymphocyte populations in all types of patients at risk of PCP rather than in a specific population such as transplanted patients. The study design could constitute a limitation, considering the diversity of immunosuppression causes that contribute to PCP risk. Nevertheless, it revealed a potential interest in several circulating lymphocyte populations and subpopulations that are rarely studied in PCP clinical studies, while confirming Zhang et al.’s observations on the protective Th1 profile in PCP found in the specific population of patients with autoimmune or inflammatory disease [12]. Furthermore, a highlight of this study is the total consistency of its observations with the reports from several murine studies. This both strengthens the veracity of these results and confirms the great similarities in host immune response to *Pneumocystis* infection between rodents and humans, and hence the usefulness of this murine model [31]. Larger and independent cohorts are now required to confirm the interest of B cells, the Th1/Tc1 profile, effector TCD8, CM TCD8 or NK cells as biomarkers in PCP, with, if possible, a sub-analysis, depending on the patient’s immunosuppression conditions.

In conclusion, this study shows the potential interest of B cell evaluation in the context of PCP. Their analysis could complement TCD4 monitoring in the assessment of PCP risk in immunocompromised patients. This study also shows that high levels of CD4 Th1 and CD8 Tc1, high NK cell counts and a low proportion of effector TCD8 could be protective against PCP severity. The traditional analysis of TCD4 and TCD8 populations may be insufficient in the context of PCP, and a deeper analysis of their subpopulations might thus be required. The use of specific thresholds which must be evaluated in larger cohorts could then help physicians predict patient outcomes. Further investigations are also necessary for understanding the mechanisms directing T helper differentiation in PCP, as a potential orientation toward a Th1 (and Tc1) response could be a possible target for improving the outcomes of patients with PCP.

## Figures and Tables

**Table 1:** Clinical and demographic characteristics of PCP positive and negative subjects.

	PCP (+)	PCP (−)	*p*-Value
n patients	20 ^a^	20 ^b^	
Median age (years) (min; max)	65 (30; 78)	58 (33; 77)	0.24
Sex ratio (M/F)	2.3 (14/6)	1.2 (11/9)	0.33
Immunosuppressive condition			
Solid organ transplant	5 (25%)	4 (20%)	1
Cancer with chemotherapy ^c^	6 (30%)	7 (35%)	0.74
Bone marrow graft	2 (10%)	2 (10%)	1
Inflammatory disease	3 (15%)	3 (15%)	1
HIV-positive	4 (20%)	4 (20%)	1
Immunosuppressive and anti-inflammatory therapies			
Corticosteroid therapy	14 (70%)	8 (40%)	0.06
2-month cumulative dose ^d^, Medians (IQR)	300 (0; 2314)	0 (0; 422)	0.04
2-month cumulative dose >3000 ^d^	5 (25%)	0 (0%)	0.05
Mofetil mycophenolate	1 (5%)	1 (5%)	1
Anticalcineurins	4 (20%)	6 (30%)	0.72
mTOR inhibitors	4 (20%)	2 (10%)	0.66
Cytotoxic agents	6 (30%)	8 (40%)	0.51
Anti-CD-20 agents	2 (10%)	2 (10%)	1
Anti-TNFα agents	0 (0%)	1 (5%)	1
Bactrim prophylaxis	0 (0%)	4 (20%)	0.11
Positive CMV PCR (blood and/or BAL)	6 (30%)	4 (20%)	0.72
Other/Co-infections	15 (75%)	12 (60%)	0.31
Bacteria	10 (50%)	9 (45%)	0.75
Viruses	10 (50%)	6 (30%)	0.2
Fungi	6 (30%)	3 (15%)	0.45
Clinical signs and hospital care			
Fever	15 (75%)	7 (35%)	0.01
Cough	12 (60%)	7 (35%)	0.11
Dyspnea	17 (85%)	9 (45%)	0.01
Ground glass or interstitial infiltrates on CT scan	18 (90%)	9 (45%)	<0.01
Intensive care admission	10 (50%)	6 (30%)	0.2
Mechanical ventilation/High-flow O2	11 (55%)	3 (15%)	0.01
2-month mortality	7 (35%)	3 (15%)	0.27

^a^: 19 subjects for TCD8 subpopulations, ^b^: 18 subjects for TCD4 and TCD8 subpopulations, ^c^: contains hematological and solid cancers, ^d^: mg of prednisone or equivalent. IQR: Interquartile range. Significant *p*-values are underlined in the table. Chi-squared test or Fisher’s exact test were performed for patient characteristics and Mann–Whitney was performed for quantitative analysis.

**Table 2:** Peripheral blood lymphocyte populations and subpopulations in PCP positive, PCP negative and control subjects.

	PCP (+)	PCP (-)		Control	
	Medians (IQR)		*p*-Value	Medians (IQR)	*p*-Value
n leukocytes (G/L)	5.5 (3.9; 12.8)	5.2 (2.5; 9.8)	0,92		
n lymphocytes (G/L)	0.9 (0.6; 1.5)	0.9 (0.4; 1.6)	0.96		
n neutrophils (G/L)	3.9 (2.3; 8.9)	4.2 (1.6; 8.4)	0.79		
n eosinophils (G/L)	0.1 (0.0; 0.2)	0.1 (0.0; 0.3)	0.49		
n basophils (G/L)	0.0 (0.0; 0.1)	0.0 (0.0; 0.1)	0.99		
n monocytes (G/L)	0.4 (0.1; 0.9)	0.6 (0.1; 1.0)	0.81		
n T lymphocytes (cells/µL)	515 (298; 1012)	551 (179; 1219)	0.99		
T lymphocytes (% lymphocytes)	67.0 (49.1; 81.9)	62.9 (40.4; 82.9)	0.69	67.0 (52.4; 81.9)	0.83
n T CD4 (cells/µL)	333 (106; 559)	331 (89; 702)	0.8		
n T CD4 < 200 cells/µL	8 (40%) ^a^	7 (35%) ^a^	0.74		
T CD4 (% CD3)	55.9 (41.8; 76.6)	73.5 (55.8; 84.9)	0.21	74.7 (67.0; 82.0)	0.14
Naive CD4 (% CD4)	24.2 (8.1; 48.9)	23.2 (4.7; 62.7)	0.9	40.5 (27.5; 51.3)	0.30
Effector CD4 (% CD4)	2.1 (1.2; 3.9)	2.2 (1.2; 4.2)	0.92	2.9 (1.7; 5.4)	0.43
Effector memory CD4 (% CD4)	29.0 (19.1; 50.1)	35.2 (12.1; 52.1)	0.69	19.9 (13.7; 34.1)	0.18
Central memory CD4 (% CD4)	30.2 (22.4; 41.9)	28.3 (23.5; 39.9)	0.8	33.9 (27.4; 41.8)	0.56
Th1 CD4 (% CD4)	12.6 (7.7; 27.4)	13.2 (7.9; 21.5)	0.78	15.6 (10.9; 20.2)	0.58
Th2 CD4 (% CD4)	0.6 (0.3; 1.2)	0.5 (0.2; 1.0)	0.36	0.3 (0.2; 0.4)	0.01 (a <0.05; b: NS)
Th17 CD4 (% CD4)	0.4 (0.1; 0.8)	0.4 (0.2; 0.7)	0.92	0.2 (0.1; 0.3)	0.03
Th1-Th17 CD4 (% CD4)	4.6 (2.0; 8.9)	4.2 (2.4; 9.4)	0.99	8.5 (5.0; 11.6)	0.04
Th9 CD4 (% CD4)	5.5 (4.1; 11.5)	7.4 (2.8; 14.6)	0.59	7.8 (6.0; 10.4)	0.55
CD4 Treg (% CD4)	5.3 (2.9; 8.2)	4.9 (3.9; 7.9)	0.7	5.4 (5.0; 6.2)	0.81
Ratio Th1/Th2	19.2 (9.1; 44.8)	21.4 (12.6; 46.3)	0.51	52.9 (29.5; 138.7)	<0.01 (a < 0.01; b < 0.05)
Ratio Th17/Treg	0.07 (0.02; 0.15)	0.06 (0.03; 0.08)	0.47	0,03 (0.01; 0.06)	0,03 (a < 0.05; b: NS)
n T CD8 (cells/µL)	117 (60; 338)	53 (31; 131)	0.13		
T CD8 (% CD3)	32.1 (15.8; 47.7)	17.1 (8.4; 25.9)	0.06	17.9 (10.9; 24.4)	0.06
Naive CD8 (% CD8)	13.5 (4.8; 33.3)	19.3 (7.0; 33.4)	0.35	19.2 (9.3; 30.8)	0.54
Effector CD8 (% CD8)	19.8 (15.6; 35.3)	30.8 (17.9; 48.4)	0.39	27.1 (18.1; 35.3)	0.55
Effector memory CD8 (% CD8)	49.3 (17.2; 75.8)	35.9 (25.1; 48.9)	0.42	41.6 (34.4; 49.9)	0.53
Central memory CD8 (% CD8)	6.0 (2.6; 10.6)	5.2 (2.9; 7.5)	0.92	8.2 (4.9; 14.1)	0.04
FoxP3+ CD8 (% CD8)	0.04 (0.00; 0.2)	0.1 (0.02; 0.4)	0.16	0.3 (0.11; 0.48)	<0.01 (a < 0.01; b: NS)
Non-naive Tc1 CD8 (% CD8)	45.5 (36.4; 70.6)	49.7 (32.0; 61.3)	0.7	57.5 (49.6; 67.0)	0.18
Non-naive Tc2 CD8 (% CD8)	1.1 (0.6; 1.9)	1.5 (1.0; 2.0)	0.32	0.8 (0.5; 1.2)	0.02
Non-naive Tc9 CD8 (% CD8)	0.3 (0.1; 0.5)	0.3 (0.1; 0.6)	0.7	0.6 (0.4; 1.5)	<0.001 (a < 0,01; b < 0.05)
Ratio CD4/CD8	1.5 (0.9; 4.7)	4.6 (2.0; 9.8)	0.1	4.0 (2.8; 7.6)	0.08
n B lymphocytes (cells/µL)	34 (6; 210)	67 (14; 220)	0.39		
n B lymphocytes <40 cells/µL	12 (60%) ^#^	5 (25%) ^#^	0.03		
B lymphocytes (% lymphocytes)	4.3 (1.2; 18.6)	12.5 (2.6; 20.9)	0.29	12.3 (7.8; 14.5)	0.21
n NK lymphocytes (cells/µL)	60 (32; 160)	65 (25; 81)	0.41		
NK lymphocytes (% lymphocytes)	7.0 (3.8; 12.4)	5.1 (2.0; 9.2)	0.3	10.2 (7.5; 15.6)	0.02 (a: NS; b < 0.05)
n NK T lymphocytes (cells/µL)	29 (11; 77)	20 (8; 39)	0.27		
NK T lymphocytes (% lymphocytes)	3.2 (1.4; 5.3)	1.8 (0.9; 7.8)	0.29	4.5 (2.5; 7.4)	0.12

^#^: number of patients below the corresponding threshold (percentage in the group). NS: not significant; IQR: Interquartile range. Significant *p*-values are underlined in the table. A Chi-squared test was performed for qualitative analysis and a Mann–Whitney U test for the quantitative analysis. A Kruskal–Wallis test was used to compare the control group and PCP(+) and PCP(−) groups, and completed with a Dunn’s test if there was a significant difference (a: Dunn’s test *p*-value for the comparison between control and the PCP(+) group, b: Dunn’s test *p*-value for the comparison between control and the PCP(−) group).

**Table 3:** Comparison of clinical characteristics and lymphocyte populations in survivors and non-survivors of the PCP(+) group.

	PCP (+)		
	Survivors	Deceased	*p*-Value
n patients	13	7 ^a^	
Median age (years) (min; max)	63 (36; 78)	68 (30; 71)	0.63
Sex ratio (M/F)	1.6 (8/5)	6 (6/1)	0.35
Immunosuppression condition			
Solid organ transplant	3 (23%)	2 (29%)	1
Cancer with chemotherapy ^b^	2 (15%)	4 (57%)	0.12
Bone marrow graft	2 (15%)	0 (0%)	0.52
Inflammatory disease	3 (23%)	0 (0%)	0.52
HIV-positive	3 (23%)	1 (14%)	1
Immunosuppressive and anti-inflammatory therapies			
Corticosteroid therapy	8 (62%)	6 (86%)	0.35
Mofetil mycophenolate	0 (0%)	1 (14%)	0.35
Anticalcineurins	3 (23%)	1 (14%)	1
mTOR inhibitors	3 (23%)	1 (14%)	1
Cytotoxic agents	2 (15%)	4 (57%)	0.12
Anti-CD-20 agents	1 (8%)	0 (0%)	1
Fungal load (30-Cq)–median (interquartile range)	7.5 (3.3; 11.2)	9.3 (4.5; 10.5)	0.75
Positive CMV PCR (blood and/or BAL)	3 (23%)	4 (57%)	0.17
Co-infections	9 (69%)	7 (100%)	0.25
Bacteria	5 (38%)	6 (86%)	0.07
Viruses	6 (46%)	4 (57%)	1
Fungi	3 (23%)	3 (43%)	0.61
Clinical signs and hospital care			
Fever	12 (92%)	4 (57%)	0.1
Cough	10 (77%)	3 (43%)	0.17
Dyspnea	11 (85%)	7 (100%)	0.52
CT scan abnormalities	12 (92%)	7 (100%)	1
Intensive care admission	5 (38%)	5 (71%)	0.35
Mechanical ventilation/High flow O2	5 (38%)	6 (86%)	0.07
	**Medians (Interquartile Ranges)**		***p*-Value**
n leukocytes (G/L)	5.4 (4.0; 10.8)	5.6 (2.9; 14.3)	1
n lymphocytes (G/L)	0.9 (0.7; 2.0)	0.6 (0.4; 0.7)	0.05
n neutrophils (G/L)	3.9 (2.5; 7.1)	4.2 (2.0; 12.4)	0.78
n eosinophils (G/L)	0.1 (0.0; 0.3)	0 (0.0; 0.1)	0.06
n basophils (G/L)	0 (0.0; 0.1)	0 (0.0; 0.1)	1
n monocytes (G/L)	0.4 (0.2; 1.1)	0.4 (0.1; 0.8)	0.32
n T CD3 (cells/µL)	620 (364; 1424)	500 (136; 538)	0.23
T CD3 (% lymphocytes)	66.4 (48.4; 79.7)	76.9 (48; 83.3)	0.58
n T CD4 (cells/µL)	363 (116; 854)	166 (89; 376)	0.2
T CD4 (% CD3)	58.6 (41; 80.2)	51.5 (42.5; 76.9)	1
Naive CD4 (% CD4)	20.2 (4.5; 41)	42.5 (24.9; 49.6)	0.11
Effectors CD4 (% CD4)	1.8 (0.7; 2.9)	2.9 (1.6; 5;7)	0.17
Effectors memory CD4 (% CD4)	31.4 (19.6; 62.6)	27.9 (19; 36.1)	0.38
Central memory CD4 (% CD4)	36.2 (23.6; 43)	28.6 (20; 39.9)	0.18
Th1 CD4 (% CD4)	19 (11.7; 30.4)	9.2 (7.0; 10.0)	0.02
Th2 CD4 (% CD4)	0.6 (0.3; 0.8)	1.3 (0.4; 1.5)	0.03
Th17 CD4 (% CD4)	0.5 (0.1; 0.8)	0.3 (0.1; 1.2)	1
Th1-Th17 CD4 (% CD4)	4.8 (3.2; 8.8)	3.9 (0.8; 9.2)	0.81
Th9 CD4 (% CD4)	6.9 (4.7; 11.7)	4.3 (2.1; 11.7)	0.34
CD4 Treg (% CD4)	6.1 (2.6; 11.1)	4.65 (3.0; 8.2)	0.81
Ratio Th1/Th2	33.3 (13.7; 108.8)	9.4 (4.3; 16.4)	0.01
Ratio Th17/Treg	0.07 (0.03; 0.13)	0.07 (0.02; 0.31)	1
n T CD8 (cells/µL)	109 (48; 599)	125 (58; 187)	0.58
T CD8 (% CD3)	29.8 (13.6; 47.7)	37.5 (16.3; 47.9)	0.94
Naive CD8 (% CD8)	13.3 (1.5; 34)	15.6 (12.3; 24.5)	0.63
Effector CD8 (% CD8)	16.5 (13.7; 23.9)	44.1 (28.6; 68.4)	<0.01
Effector memory CD8 (% CD8)	51.3 (26.1; 76.7)	24.5 (15.4; 43.3)	0.1
Central memory CD8 (% CD8)	6.4 (4.1; 11.8)	2.3 (1.2; 7)	0.04
CD8 Treg (% CD8)	0.03 (0.0; 0.2)	0.07 (0.02; 0.2)	0.93
Non-naive Tc1 CD8 (% CD8)	58.7 (45.2; 71.8)	36.0 (29.9; 45.5)	0.03
Non-nave Tc2 CD8 (% CD8)	1.1 (0.9; 1.6)	1.2 (0.5; 2.4)	0.97
Non-naive Tc9 CD8 (% CD8)	0.2 (0.1; 0.4)	0.3 (0.1; 0.5)	0.57
Ratio CD4/CD8	1.7 (0.9; 6)	1.4 (0.9; 4.7)	1
n B lymphocytes (cells/µL)	42 (6; 321)	25 (5; 55)	0.43
B lymphocytes (% lymphocytes)	4.7 (1.3; 19.9)	3.2 (0.9; 7.9)	0.53
n NK lymphocytes (cells/µL)	94 (51; 165)	31 (6; 45)	0.02
NK lymphocytes (% lymphocytes)	9.2 (5.0; 24)	4.5 (1.0; 7.1)	0.1
n NK T lymphocytes (cells/µL)	37 (12; 82)	22 (8; 55)	0.63
NK T lymphocytes (% lymphocytes)	3.2 (1.3; 5.3)	3.3 (1.6; 9.1)	0.69

^a^: 6 patients for TCD8 subpopulations, ^b^: contains hematological and solid cancers. Significant *p*-values are underlined in the table. Chi-squared test or Fisher’s exact test was performed for patient characteristics; Mann–Whitney U test was performed for quantitative lymphocyte parameters).

**Table 4:** Results of ROC curve analysis.

	Th1/Th2	% Tc1 CD8	% Effector CD8	% CM CD8	n NK Cells	n Lymphocytes
Threshold	17	44%	25%	4%	50 cells/µL	0.75 G/L
Sensitivity (%)	85.7	83.3	100	83.3	85.7	85.7
Specificity (%)	83.3	83.3	83.3	83.3	75	75
PPV (%)	75	71.4	75	71.4	66.7	66.7
NPV (%)	90.9	90.9	100	90.9	90	90
AUC (%)	88.1	83.3	93.1	81.3	82.1	76.8

CM: central memory.

## Data Availability

Not applicable.

## Supplementary Materials

The following are available online at https://www.mdpi.com/article/10.3390/jof7080652/s1, Figure S1: Gating strategy for lymphocyte immunophenotyping, Figure S2: ROC curves for mortality risk, Figure S3: Lymphocyte subpopulations according to total blood lymphocyte count threshold (0.75 G/L), Table S1: Comparison of the area under the curve (AUC) for the ROC analysis.
