# Supplementary material for: New Insights into Blood Circulating Lymphocytes in Human Pneumocystis Pneumonia
Source: J Fungi (Basel). 2021 Aug 11;7(8):652. doi: 10.3390/jof7080652 (PMC8400826; doi:10.3390/jof7080652)
Supplement: Supplementary file 1 [file jof-07-00652-s001.zip › jof-1285447-supplementary.pdf]

SUPPLEMENTARY DATA

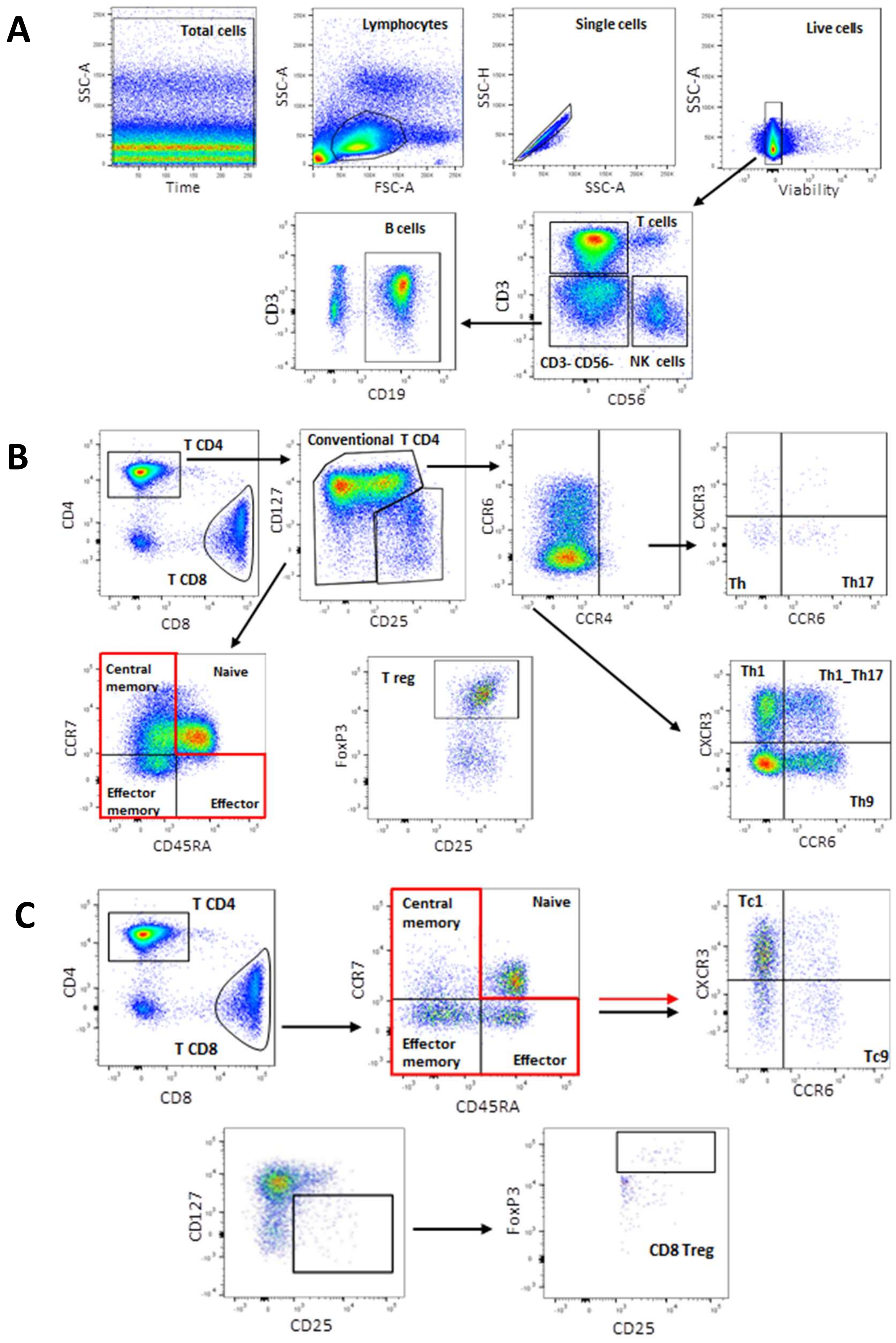

Supplemental Figure S1: Gating strategy for lymphocyte immunophenotyping.

A: general lymphocyte immunophenotyping. B: CD4 subpopulation gating from T cells determined in gating strategy A. C: CD8 subpopulation gating from T cells determined in gating strategy A.

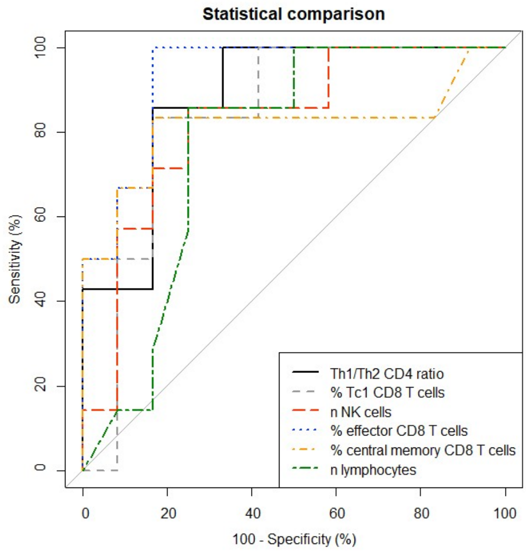

Supplemental Figure S2: ROC curves for mortality risk

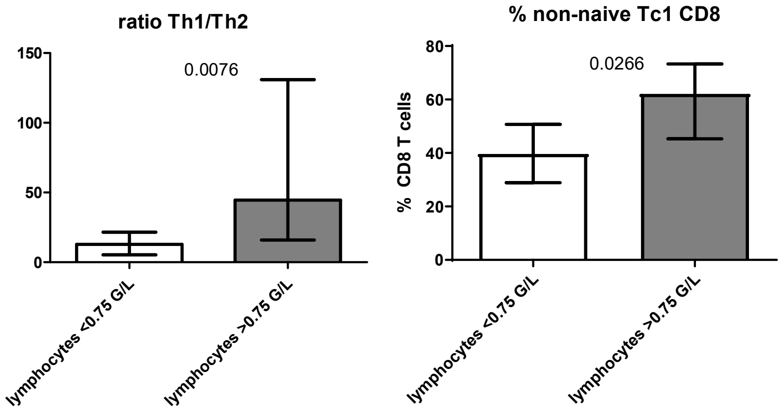

Supplemental Figure S3: Lymphocyte subpopulations according to total blood lymphocyte count threshold (0.75 G/L).

Data are expressed as medians [interquartile intervals] and compared by the Mann-Whitney U test. P-values are added on each graph.

		Th1/Th2	% Tc1 CD8	n NK cells	% effector CD8	% CM CD8	n lymphocytes
	AUC	88.1%	83.33%	82.14%	93.06%	81.25%	76.79%
Th1/Th2	88.1%	-	p= 0.34	p=0.65	p=0.56#	p=0.80	p=0.36
% Tc1 CD8	83.33%		-	p=0.86	p=0.44#	p=0.90	p=0.63
n NK cells	82.14%			-	p=0.32#	p=0.91	p=0.61
% effector CD8	93.06%				-	p=0.37#	p=0.19#
% central memory CD8	81.25%					-	p=0.70
n lymphocytes	76.79%						-

Supplemental Table S1: Comparison of the area under the curve (AUC) for the ROC analysis.

AUC: Area under the curve. #: comparison performed with Bootstrap test. There was no significant difference between the AUCs (p >0.05).
